# Relationship between depression and burnout among nurses in Intensive Care units at the late stage of COVID-19: a network analysis

**DOI:** 10.1186/s12912-024-01867-3

**Published:** 2024-04-01

**Authors:** Yinjuan Zhang, Chao Wu, Jin Ma, Fang Liu, Chao Shen, Jicheng Sun, Zhujing Ma, Wendong Hu, Hongjuan Lang

**Affiliations:** 1 Department of Nursing, Air Force Medical University, No. 169 Changle West Road, 710032 Xi’an, Shaanxi China; 2 Department of Nursing, Shaanxi University of Chinese Medicine, Shiji Avenue, 712046 Xianyang, Shaanxi China; 3 Department of Aerospace Medicine, Air Force Medical University, No. 169 Changle West Road, 710032 Xi’an, Shaanxi China; 4 Department of Computer Science and Engineering, Xi’an Technological University, No. 4 Jinhua North Road, 710021 Xi’an, Shaanxi China; 5 Department of Military Medical Psychology, Air Force Medical University, No. 169 Changle West Road, 710032 Xi’an, Shaanxi China

**Keywords:** Burnout, Depression, Network analysis, ICU nurses

## Abstract

**Background:**

Mental health problems are critical and common in medical staff working in Intensive Care Units (ICU) even at the late stage of COVID-19, particularly for nurses. There is little research to explore the inner relationships between common syndromes, such as depression and burnout. Network analysis (NA) was a novel approach to quantified the correlations between mental variables from the perspective of mathematics. This study was to investigate the interactions between burnout and depression symptoms through NA among ICU nurses.

**Method:**

A cross-sectional study with a total of 616 Chinese nurses in ICU were carried out by convenience sampling from December 19, 2022 to January19, 2023 via online survey. Burnout symptoms were measured by Maslach Burnout Inventory-General Survey (MBI-GS) (Chinese version), and depressive symptoms were assessed by the 9-item Patient Health Questionnaire (PHQ-9). NA was applied to build interactions between burnout and depression symptoms. We identified central and bridge symptoms by R package qgraph in the network model. R package bootnet was used to examined the stability of network structure.

**Results:**

The prevalence of burnout and depressive symptoms were 48.2% and 64.1%, respectively. Within depression-burnout network, PHQ4(Fatigue)-MBI2(Used up) and PHQ4(Fatigue)-MBI5(Breakdown) showed stronger associations. MBI2(Used up) had the strongest expected influence central symptoms, followed by MBI4(Stressed) and MBI7 (Less enthusiastic). For bridge symptoms. PHQ4(Fatigue), MBI5(Breakdown) and MBI2(Used up) weighed highest. Both correlation stability coefficients of central and bridge symptoms in the network structure were 0.68, showing a high excellent level of stability.

**Conclusion:**

The symptom of PHQ4(Fatigue) was the bridge to connect the emotion exhaustion and depression. Targeting this symptom will be effective to detect mental disorders and relieve mental syndromes of ICU nurses at the late stage of COVID-19 pandemic.

**Supplementary Information:**

The online version contains supplementary material available at 10.1186/s12912-024-01867-3.

## Introduction

Since COVID-19 broke out in Wuhan of China in December 2019, the World Health Organization (WHO) on March 11, 2020 categorized the disease as a worldwide epidemic as global rapidly spreading [1, 2]. People hit by SARS-CoV-2 were more prone to develop acute respiratory distress syndrome (ARDS) or even multiple system organ failure. Studies reported approximately 5–10% of patients diagnosed with COVID-19 were admitted to Intensive Care Units (ICU) for critical care due to the high mortality [3, 4].

With the use of vaccines and the implementation of active epidemic prevention measures in China, the nationwide lockdown policy was ended. China entered the late stage of the COVID-19 pandemic in April 2020, and the China National Health Commission (CNHC) announced to lift most of the restrictions implemented for “Zero-COVID” policy (restricting mass gatherings, maintaining social distancing, and staying at home) on December 7, 2022 [5]. The pandemic of China came to a peak stage again in a short time and the number of COVID-19 patients in the ICUs was increasing rapidly due to high-speed spreading. ICU, unlike other parts of the hospital, are areas where complex and state-of-the-art devices are used and special treatment and care is delivered. Nurses in ICU are the backbones of effective health systems during this pandemic [6]. ICU nurses were confronted with difficult conditions, such as substantial workload, prolonged work hours and considerable risk of infection, which led to serious mental distress [7, 8] and resulted in an increasing risk of psychiatric health problems, such as depression and burnout [9, 10].

Many studies reported that nurses working in ICU showed high risk of depression during the pandemic of COVID-19 [11–13]. A cross-sectional survey demonstrated over 40% of ICU nurses suffered from moderate to severe symptoms of depression during COVID-19 [14]. One systematic and meta-analysis including 20,617 healthcare workers proved that the prevalence of burnout in ICU nurses achieved 45% [15]. Burnout and depression of nurses had adverse effects on health of patients, and even threatened the safety of patient (i.e., medical administration errors, injury even death) [16–18], which was a global healthcare concern [19]. Besides, the intention to leave work in ICU nurses was rising during COVID-19. The WHO predicted that there would be a deficit of around 7.6 million nurses globally by 2030 before COVID-19 [20]. This deficit seemed likely to be greater as recent research had indicated that about 20% nurses had a thoughtful consideration of quitting for adverse mental health outcomes. Between them, ICU nurses had the highest of nearly 27% [21], which would be detrimental to the sustainable development of the nursing profession. Therefore, efforts should be made to improve the burnout and depression for this important group of care providers.

WHO in 2022 announced the implement of 7th of the International Classification of Disease [22], in which burnout is defined as a syndrome caused by chronic occupational stress that has not been managed successfully, and has resulted in feeling of energy exhaustion, negativism related to one’s work and lack of achievement. Nurse engaging in ICU are particular prone to suffer burnout due to exposure to pain, trauma, dying and closed environments, not necessary within pandemic [23]. Depression is a leading cause of disability and contributes greatly to global burden of disease [24]. People suffered from depression are characterized by persisted sadness, diminished pleasure or interest, even feeling of excessive guilt, hopelessness. Severe depression patients will think of self-harm or suicide [22]. WHO in 2022 has listed depression as one of high-risk factors leading to disability and the major contributor to suicide [24]. Depression not only negatively impact well-being of ICU nurses, but also extract toll on the health industry, with a severe adverse effect on healthcare quality [25, 26].

The relation between burnout and depression has received a great deal of attention in recent years [27, 28]. Burnout was identified as one of strongest predictor of depressive symptoms [29, 30]. Meanwhile, depression symptoms contributed to the development of burnout [31]. But more importantly, burnout often co-occurred with depression [32]. A system review and meta-analysis reported that over 50% employers with burnout had depression [33]. A survey of healthcare workers in Macao and China found that depression was associated with all subscales of burnout after controlling for the strong effects of demographic factors [34]. 16.5% of psychiatric with depressive symptoms had high rate of burnout [35]. These data suggest there exist strong associations between burnout and depression symptoms. But how the symptoms of the two variables are associated still remain unclear.

Altogether, the previous studies have explored the interactions between burnout and depression at the syndrome level using traditional correlational methods, which included path analysis and multiple regression analysis in general. But those methods were based on the assumption of linear relationship, and couldn’t describe the complex non-linear contact between burnout and depression symptoms [36]. In order to overcome the issue, network analysis (NA) was applied to quantify the correlations between burnout and depression symptoms from the perspective of mathematical and display it intuitively. It wasn’t just on the basis of assumptions but a data-driven approach about causality between multiple variables [37]. In the theory of NA, mental syndromes and disorders were induced by the direct interactions between their corresponding symptoms, which included nodes representing observed variables (e.g., 15 nodes of burnout and 10 nodes of depression) and edges representing the associations between nodes. Therefore, exploring the accurate interactions was critical to elaborate psychopathological mechanisms and develop targeted intervention policies. Furthermore, NA could also provide centrality and predictability indices of each node, which helped researchers to identify and quantify to what extent burnout may transmit positive/negative influence to depression [38]. Since central symptoms in a network model closely connected with other symptoms, and they might active other symptoms. Thus, central symptoms with higher ranking score might become the target of treatment interventions, as they had a significant impact on the network. NA provided a new way to understand human psychological phenomena, and had been applied to the research of social psychology, clinical psychology, psychiatry and other fields [39, 40].

Several studies have explored the symptom level interactions between burnout and depression symptoms using NA among different groups of people. Network structure among educational professions demonstrated that suicidal thoughts was only associated with other symptoms of depression, but not with those of burnout [41]. Another study showed that the symptoms of “feel down-hearted” and “no hope for future” were target interventions to relieve mental disorders of pharmacists [42]. However, it was uncertain whether these findings could be generalized to ICU nurses. Therefore, the current study applied the NA to further examine the interrelationship between burnout and depression symptom of ICUs nurses in order to implement effective and targeted interventions to prevent or reduce the occurrence of burnout and depression. The aims of the current study were two-fold: (1) to explore potential pathways linking between burnout and depression symptoms; (2) to use bridge expected influence to identify the most influential symptoms within the burnout-depression network.

## Method

### Participants

A cross-sectional study was conducted among ICU nurses from December, 19 in 2022 to February, 19 in 2023 across six hospitals, which were Grade III-A General Hospitals of Shaanxi province of China. 616 nurses took part in the study. Due to COVID-19 pandemic, face-to-face assessment were not adapted. Following the previous researches during pandemic [43, 44], the WeChat-based “Questionnaire Star” program was applied to conduct online survey. WeChat is a social media for communication, which has been used widely from 2017. The users now have achieved over 1.2 billion in China. Participants met the following inclusion criteria:(1) aged 18 and older; (2) be registered nurses who worked longer than 1 year in ICU; (3) engaged in frontline clinical nursing; (4) cared patients with COVID-19. Participants who were nursing students or had mental or physical disease were excluded from the study to ensure the integrity of the study’s outcomes. The study had met with the approval of the Ethics Committee of the Second Affiliated Hospital of Shaanxi University of Chinese Medicine (No. SZFYIEC-YJ-2020-38). All participants were voluntary to join in this study and signed the informed consent form.

In order to ensure the effectiveness of online survey, we contacted with head nurses of ICU in advance and made them know the inclusion and exclusion criteria of our study clearly before the investigation. Then they send the online survey link to nurses who satisfied the requirements of our study. At last, we checked the answers of all the participants and deleted questionnaires with missing items after the survey. Besides, the participants would get a random lucky money to thank for their participation. A total of 636 nurses completed the survey, and 20 participants missed some items of questionnaire and demographic information. The effective rate was 97%.

### Measures

Maslach Burnout Inventory-General Survey (MBI-GS) (Chinese version) was used to measure the severity of burnout symptoms [45]. The 15 items of MBI-GS were scored on a seven-point Likert scale from “0” (never) to “6” (every day) capturing three dimensions: emotional exhaustion, cynicism and reduced personal achievement, with higher total scores indicating higher level of burnout. MBI-GS has extensively been applied to assess the mental distress in healthcare workers [46]. A sum score of MBI-GS above 34 was considered as suffering from burnout. The reliability of MBI-GS was evidenced in this study with a Cronbach’s alpha of 0.845. Depression was assessed by Chinese version of Patient Health Questionnaire (PHQ-9), which included 9 items with each scored on a four-Likert scale from “0” (not at all) to “3” (nearly every day). Higher scores of PHQ-9 indicated more severe depression symptoms. PHQ-9 gained strong validity and was widely used in the Chinese population [47]. Clinically relevant symptoms of depression were indicated by total score of 5 or higher on the PHQ-9 [48]. The reliability of PHQ-9 was evidenced in this study with a Cronbach’s alpha of 0.820.

### Statistical analysis

#### Network estimation

The network of burnout and depressive symptoms was constructed by R software [49]. The polychoric correlations (i.e., edges) between all the MBI-GS and PHQ-9 items, were calculated based on the Graphical Gaussian Model (GGM) with the graphic least absolute shrinkage and selection operator (LASSO) and Extended Bayesian Information Criterion (EBIC) mode [50], and the R package qgraph was used to visualize the network model [51]. The edge color of blue indicated that the connection was positive, and red was negative. Besides, the edge thickness and saturation indicated connection strength. The stronger the connection, the thicker the edge, and the more saturated it was. We also calculated the central index expected influence (EI) by R package qgraph to identify the significance of each node in the network [52]. Nodes showing higher EI were considered to be more important in the network model. The bridge expected influence (BEI) of each item was calculated to identify bridge node that linked the burnout and depression in the current study [53], which represented the importance of one symptom linking two clusters of psychiatric symptoms [37]. In addition to, the package mgm was used to check the predictability of each node, which indicated the variance in a node that was affected by other nodes connected to it.

#### Network stability

In order to estimate the accuracy of the network model, R package bootnet was used to check the stability of EI and BEI [51]. The accuracy of the edge weight value was tested by calculating its estimated confidence interval (95% CI). The stability of IE and BIE were assessed by computing the correlation stability coefficients (CS-C). In general, the CS-C above 0.5 was ideal and should not be below 0.25 [51]. In order to check the difference between edge weights and node expected influence, bootstrapped difference tests were also conducted.

## Results

### Study sample

A total of 616 ICU nurses completed the study (Table 1). The majority of the participants were female (490, 79%). The mean age was 28.0 ± 8.37 years, and the average number of working hours was 3.2 ± 0.65 years. The prevalence of burnout and depressive symptoms were 48.2% and 64.1%, respectively. Mean scores of the burnout and depression items with their SDs, expected influence, and predictability were shown in Table 2.

**Table 1:** Demographic characteristics of the participants

Characteristics	Variables	N (%)
Age	18∼30	415 (67.4%)
	31∼40	150 (24.3%)
	≥ 41	51 (8.3%)
Gender	Female	490 (79.6%)
	Male	126 (20.4%)
Marriage	Married	386(62.7%)
	Single or divorced	230 (37.3%)
Education level	Junior college	179 (29.1%)
	Bachelor degree	425(69.0%)
	Master degree	12 (1.9%)
Working years	1∼5	325 (52.8%)
	6∼10	163(26.5%)
	≥ 11	128 (20.7%)
Professional title	Junior	226(36.7%)
	Middle	310 (50.3%)
	Senior	80(13.0%)
Prevalence of burnout symptoms (Standard score > 34)		297(48.2%)
Prevalence of Depression symptoms (Standard score > 5)		395 (64.1%)

**Table 2:** Average scores of items in burnout and depression network(*N* = 616)

Item abbreviation	Item content	M	SD	Expected Influence	Predictability
MBI1	Exhausted	2.63	1.50	0.501	0.749
MBI2	Used up	2.82	0.59	0.462	0.786
MBI3	Tired	2.39	1.55	0.528	0.721
MBI4	Stressed	2.36	1.55	0.454	0.793
MBI5	Breakdown	1.83	0.43	0.552	0.695
MBI6	Less interested	1.73	0.47	0.462	0.787
MBI7	Less enthusiastic	1.70	0.51	0.477	0.772
MBI8	Doubt significance	1.45	0.52	0.451	0.796
MBI9	Indifferent	1.45	0.51	0.47	0.778
MBI10	Effective	2.42	1.61	0.618	0.617
MBI11	Contributing	2.34	1.05	0.55	0.697
MBI12	Good at job	2.22	1.14	0.637	0.594
MBI13	Happy	2.24	1.08	0.523	0.726
MBI14	Worthwhile	2.39	1.10	0.532	0.717
MBI15	Confident	2.21	1.58	0.579	0.665
PHQ1	Anhedonia	0.86	0.45	0.521	0.728
PHQ2	Sad mood	0.79	0.36	0.577	0.666
PHQ3	Sleep	1.10	0.41	0.728	0.469
PHQ4	Fatigue	1.06	0.39	0.58	0.663
PHQ5	Appetite	0.85	0.28	0.652	0.575
PHQ6	Guilty	0.65	0.31	0.568	0.676
PHQ7	Concentration	0.73	0.42	0.622	0.613
PHQ8	Motor	0.54	0.28	0.617	0.619
PHQ9	Suicide	0.20	0.13	0.905	0.28

M, Mean, SD, standard deviation

### Network structure

**Fig. 1:**
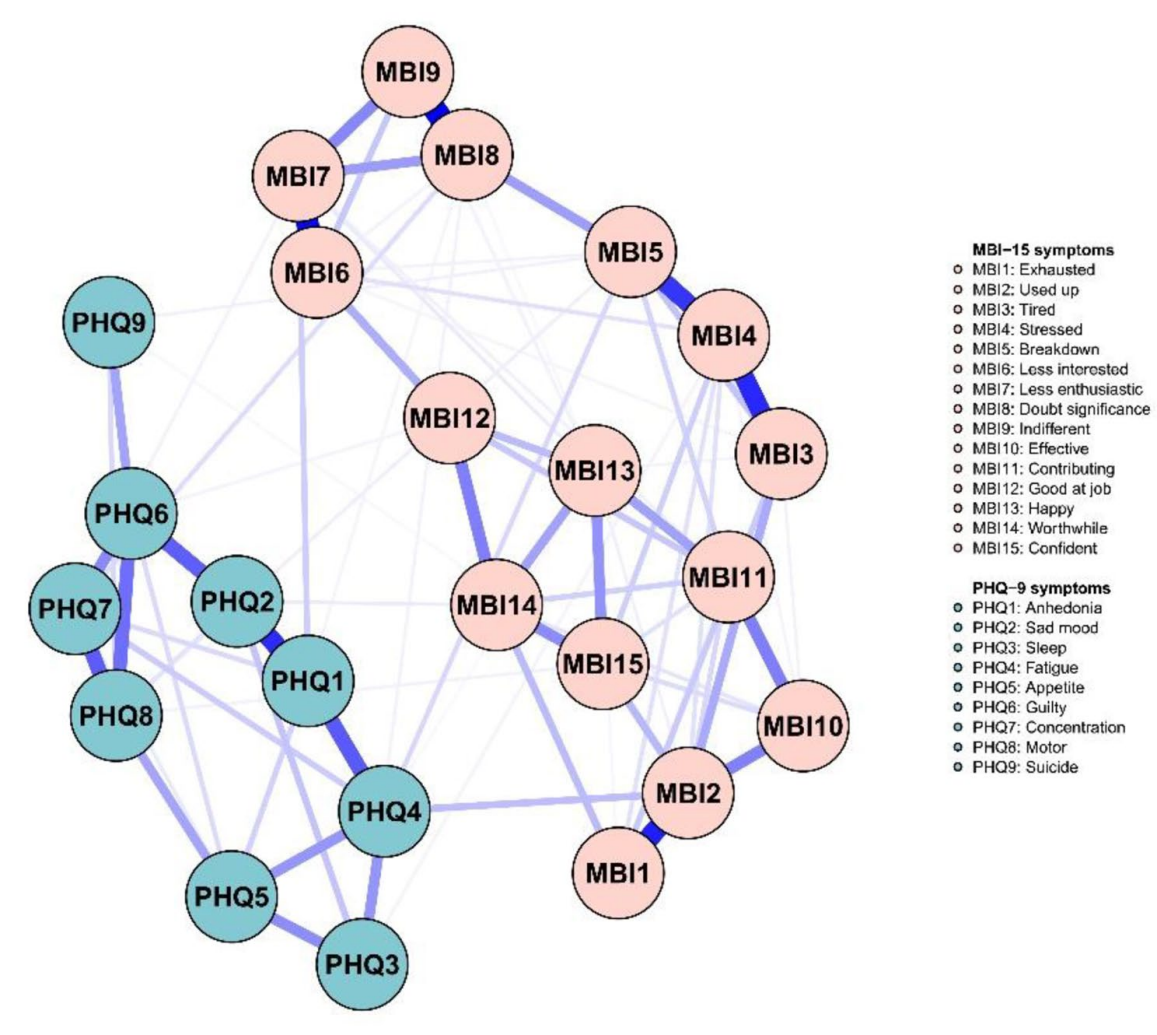
Network structure of burnout-depressive symptoms

Figure 1 showed the network model of burnout and depression symptoms, and all the edges were positive. In the burnout symptoms, the strongest edge was MBI8 (Doubt significance)-MBI9 (Indifferent), followed by the edges MBI6 (Less interested)-MBI7 (Less enthusiastic) and MBI1 (Exhausted)-MBI2 (Used up). In the PHQ-9 symptoms, the strongest edge was PHQ2 (Sad mood)-PHQ1 (Anhedonia), followed by edges PHQ4 (Fatigue)-PHQ1 (Anhedonia) and PHQ8 (Motor)-PHQ7 (Concentration).

In the burnout-depression network, the association between PHQ4 (Fatigue)- MBI2 (Used up) was the strongest, followed by PHQ1 (Anhedonia)-MBI7 (Less enthusiastic), and PHQ4 (Fatigue)-MBI5 (Breakdown) (Table 3). Table 3 showed the strength of each edge. Furthermore, the predictability of each node was showed, ranging from 0.28 to 0.79 with average value of 0.67 (Table 2).

**Table 3:** Correlation matrix of the burnout and depression items

	MBI1	MBI2	MBI3	MBI4	MBI5	MBI6	MBI7	MBI8	MBI9	MBI10	MBI11	MBI12	MBI13	MBI14	MBI15	PHQ1	PHQ2	PHQ3	PHQ4	PHQ5	PHQ6	PHQ7	PHQ8	PHQ9
MBI1		0.406	0.096	0.058	0.000	0.000	0.000	0.000	0.000	0.000	0.020	0.000	0.017	0.124	0.000	0.000	0.000	0.000	0.000	0.000	0.000	0.000	0.000	0.000
MBI2	0.041		0.153	0.059	0.000	0.000	0.000	0.000	0.000	0.226	0.073	0.000	0.028	0.000	0.129	0.000	0.000	0.000	0.117	0.000	0.000	0.000	0.000	0.000
MBI3	0.096	0.153		0.386	0.110	0.020	0.000	0.000	0.000	0.024	0.000	0.000	0.022	0.000	0.000	0.000	0.000	0.000	0.000	0.000	0.000	0.000	0.000	0.000
MBI4	0.058	0.059	0.386		0.362	0.054	0.000	0.000	0.000	0.019	0.000	0.000	0.000	0.000	0.087	0.000	0.000	0.022	0.000	0.000	0.000	0.000	0.000	0.000
MBI5	0.000	0.000	0.110	0.362		0.025	0.000	0.175	0.000	0.000	0.079	0.000	0.000	0.000	0.000	0.000	0.000	0.000	0.070	0.000	0.000	0.000	0.031	0.028
MBI6	0.000	0.000	0.020	0.054	0.025		0.447	0.023	0.102	0.000	0.000	0.146	0.000	0.000	0.000	0.000	0.000	0.000	0.000	0.000	0.000	0.000	0.000	0.000
MBI7	0.000	0.000	0.000	0.000	0.000	0.447		0.179	0.217	0.000	0.028	0.000	0.037	0.000	0.000	0.090	0.000	0.000	0.000	0.000	0.000	0.024	0.000	0.000
MBI8	0.000	0.000	0.000	0.000	0.175	0.023	0.179		0.460	0.000	0.000	0.000	0.015	0.000	0.000	0.020	0.000	0.025	0.000	0.000	0.000	0.062	0.000	0.000
MBI9	0.000	0.000	0.000	0.000	0.000	0.102	0.217	0.460		0.000	0.000	0.000	0.026	0.000	0.000	0.000	0.000	0.000	0.000	0.000	0.000	0.000	0.000	0.000
MBI10	0.000	0.226	0.024	0.019	0.000	0.000	0.000	0.000	0.000		0.231	0.000	0.000	0.048	0.065	0.000	0.000	0.000	0.000	0.000	0.000	0.000	0.000	0.000
MBI11	0.020	0.073	0.000	0.000	0.079	0.000	0.028	0.000	0.000	0.231		0.104	0.169	0.085	0.056	0.000	0.000	0.000	0.000	0.000	0.000	0.000	0.000	0.000
MBI12	0.000	0.000	0.000	0.000	0.000	0.146	0.000	0.000	0.000	0.000	0.104		0.122	0.230	0.000	0.000	0.000	0.000	0.000	0.000	0.022	0.000	0.000	0.000
MBI13	0.017	0.028	0.022	0.000	0.000	0.000	0.037	0.015	0.026	0.000	0.169	0.122		0.165	0.195	0.000	0.000	0.000	0.012	0.000	0.000	0.000	0.000	0.000
MBI14	0.124	0.000	0.000	0.000	0.000	0.000	0.000	0.000	0.000	0.048	0.085	0.230	0.165		0.208	0.000	0.037	0.000	0.000	0.000	0.000	0.000	0.000	0.000
MBI15	0.000	0.129	0.000	0.087	0.000	0.000	0.000	0.000	0.000	0.065	0.056	0.000	0.195	0.208		0.000	0.000	0.000	0.000	0.000	0.000	0.000	0.025	0.014
PHQ1	0.000	0.000	0.000	0.000	0.000	0.000	0.090	0.020	0.000	0.000	0.000	0.000	0.000	0.000	0.000		0.381	0.000	0.297	0.083	0.000	0.069	0.000	0.000
PHQ2	0.000	0.000	0.000	0.000	0.000	0.000	0.000	0.000	0.000	0.000	0.000	0.000	0.000	0.037	0.000	0.381		0.107	0.000	0.000	0.288	0.000	0.050	0.000
PHQ3	0.000	0.000	0.000	0.022	0.000	0.000	0.000	0.025	0.000	0.000	0.000	0.000	0.000	0.000	0.000	0.000	0.107		0.192	0.206	0.000	0.000	0.000	0.000
PHQ4	0.000	0.117	0.000	0.000	0.070	0.000	0.000	0.000	0.000	0.000	0.000	0.000	0.012	0.000	0.000	0.297	0.000	0.192		0.189	0.000	0.093	0.000	0.000
PHQ5	0.000	0.000	0.000	0.000	0.000	0.000	0.000	0.000	0.000	0.000	0.000	0.000	0.000	0.000	0.000	0.083	0.000	0.206	0.189		0.066	0.040	0.162	0.000
PHQ6	0.000	0.000	0.000	0.000	0.000	0.000	0.000	0.000	0.000	0.000	0.000	0.022	0.000	0.000	0.000	0.000	0.288	0.000	0.000	0.066		0.211	0.262	0.164
PHQ7	0.000	0.000	0.000	0.000	0.000	0.000	0.024	0.062	0.000	0.000	0.000	0.000	0.000	0.000	0.000	0.069	0.000	0.000	0.093	0.040	0.211		0.293	0.000
PHQ8	0.000	0.000	0.000	0.000	0.031	0.000	0.000	0.000	0.000	0.000	0.000	0.000	0.000	0.000	0.025	0.000	0.050	0.000	0.000	0.162	0.262	0.293		0.072
PHQ9	0.000	0.000	0.000	0.000	0.028	0.000	0.000	0.000	0.000	0.000	0.000	0.000	0.000	0.000	0.014	0.000	0.000	0.000	0.000	0.000	0.164	0.000	0.072	

MBI1- Exhausted, MBI2-Used up, MBI3- Tired, MBI4- Sressed, MBI5- Breakdown, MBI6- Less interested, MBI7- Less enthusiastic, MBI8-Doubt significance; MBI9-Indifferent; MBI10-Effective; MBI11-Contributing; MBI12-Good at job; MBI13-Happy; MBI14-Worthwhile; MBI15-Confident.

PHQ1- Anhedonia, PHQ2-Sad mood, PHQ3-Sleep, PHQ4-Fatigue, PHQ5- Appetite, PHQ6- Guilty, PHQ7-Concentration, PHQ8-Motor, PHQ9-Suicide.

### Network stability

**Fig. 2:**
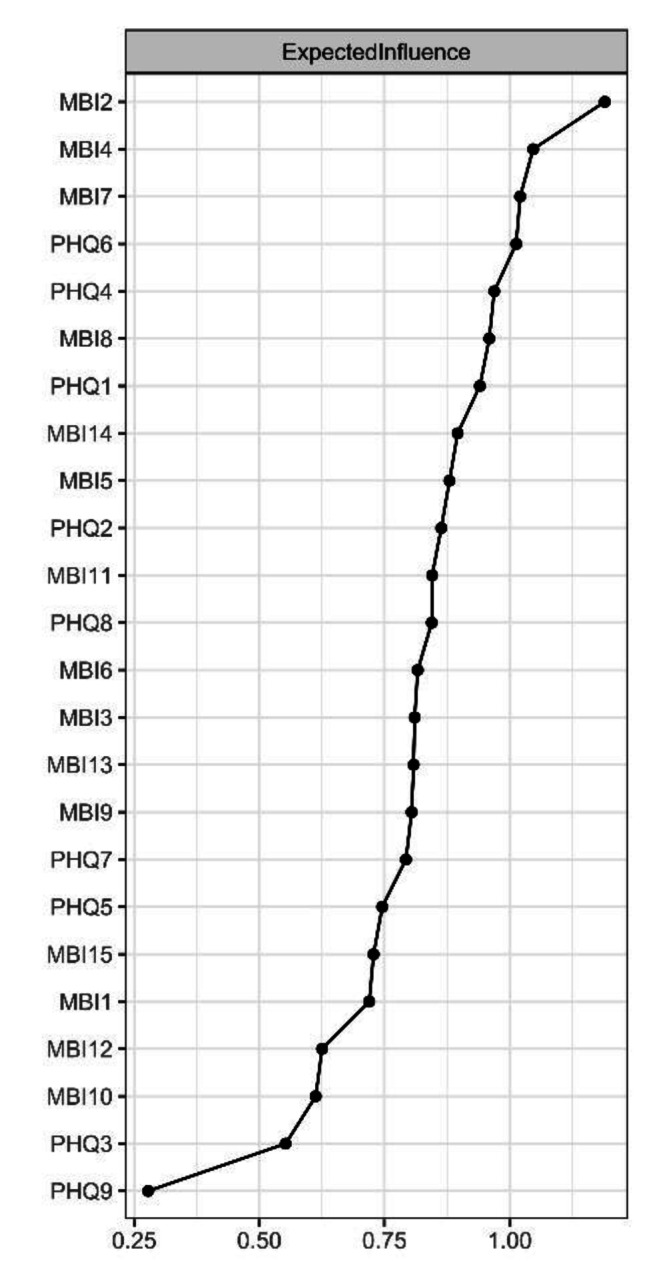
The node expected influence plot. The X-rays represented the expected influence of each node

**Fig. 3:**
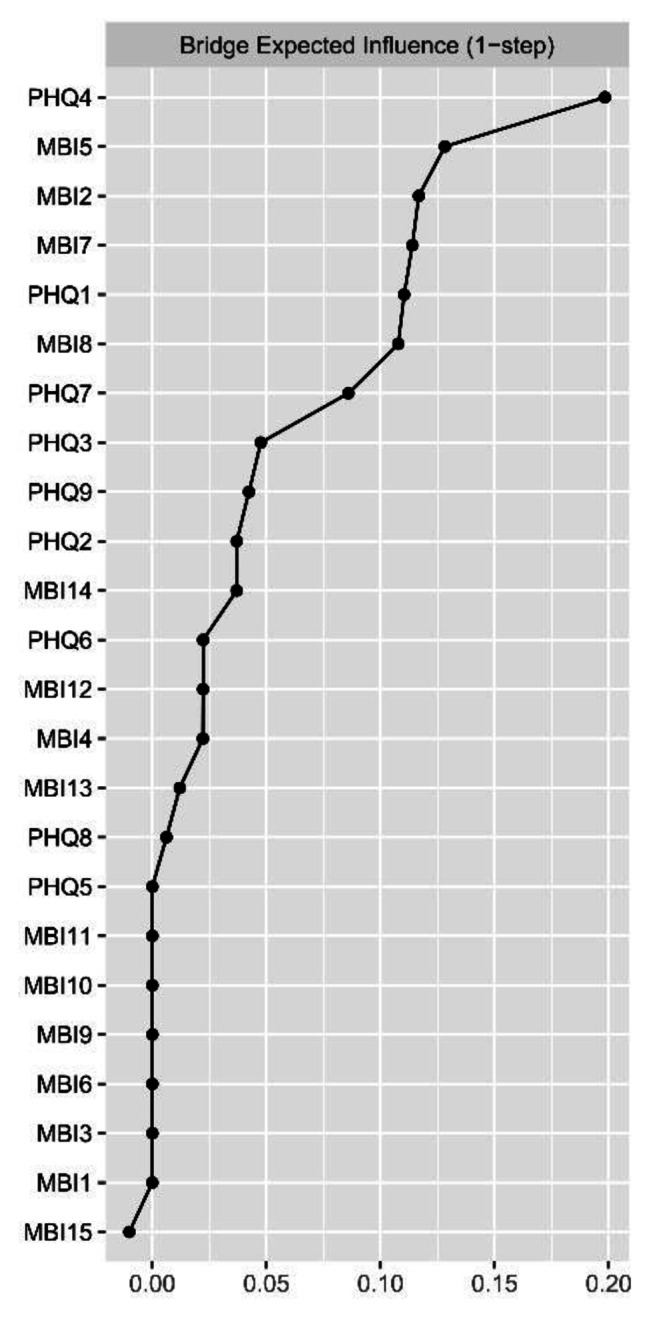
The bridge expected influence plot. The X-rays represented the bridge expected influence of each node

For centrality index expected influence (EI) (Fig. 2; Table 2), the node MBI2 (Used up) had the highest EI value, followed by MBI4 (Stressed), MBI7 (Less enthusiastic), PHQ6 (Guilty) and PHQ4 (Fatigue), implying that these symptoms were the central and influential for effecting the network model of burnout and depression among ICUs, whereas PHQ9 (Suicide) and PHQ3 (Sleep) had lowest EI value, showing marginal effect with the network. For bridge expected influence (BEI) (Fig. 3), PHQ4 (Fatigue) had the highest BEI value, followed by MBI5 (Breakdown), MBI2 (Used up), MBI7 (Less enthusiastic) and PHQ1 (Anhedonia), indicating these symptoms linking the burnout and depression symptoms at the late stage of COVID-19.

**Fig. 4:**
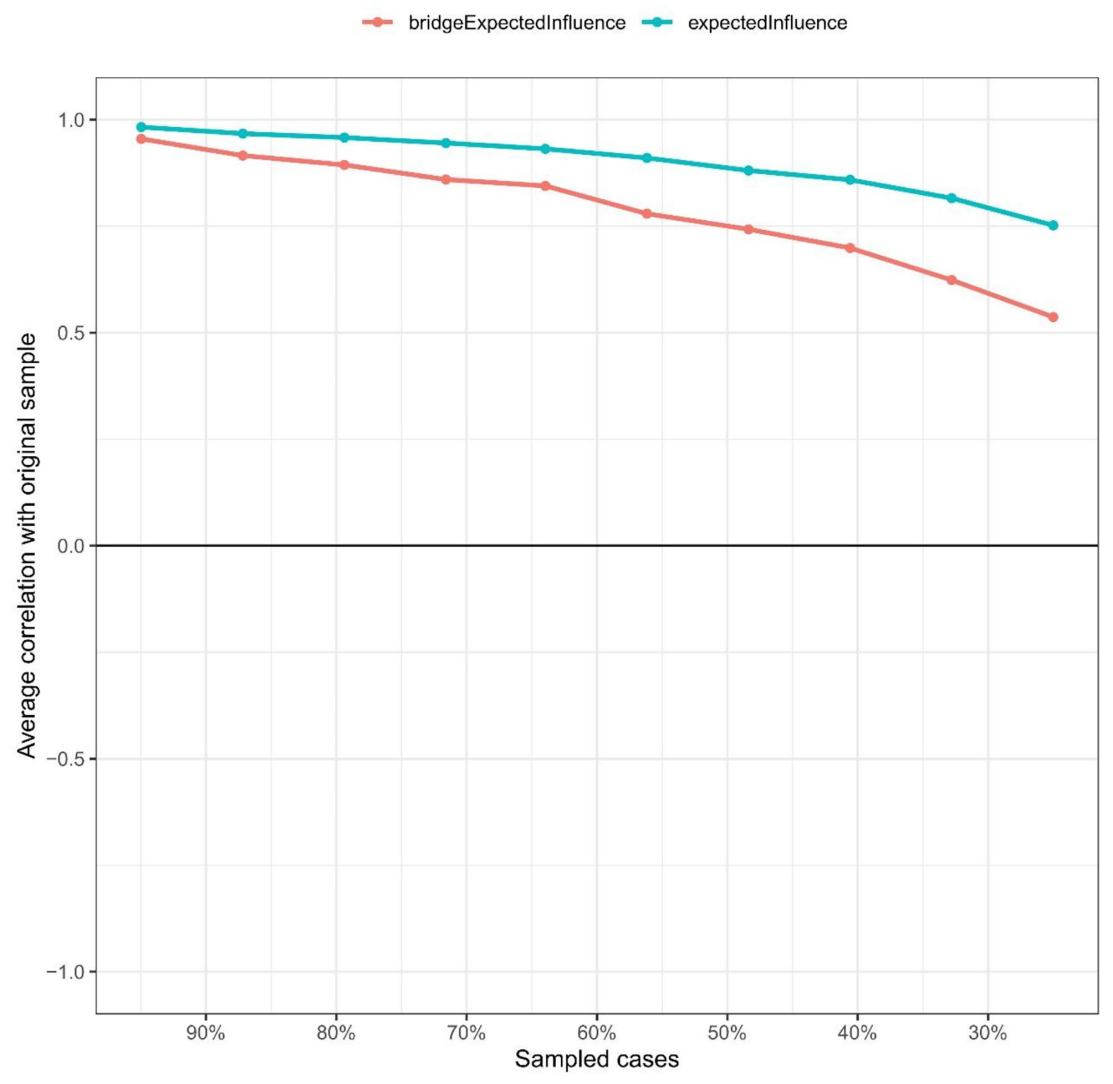
The stability of the burnout- depression network

**Fig. 5:**
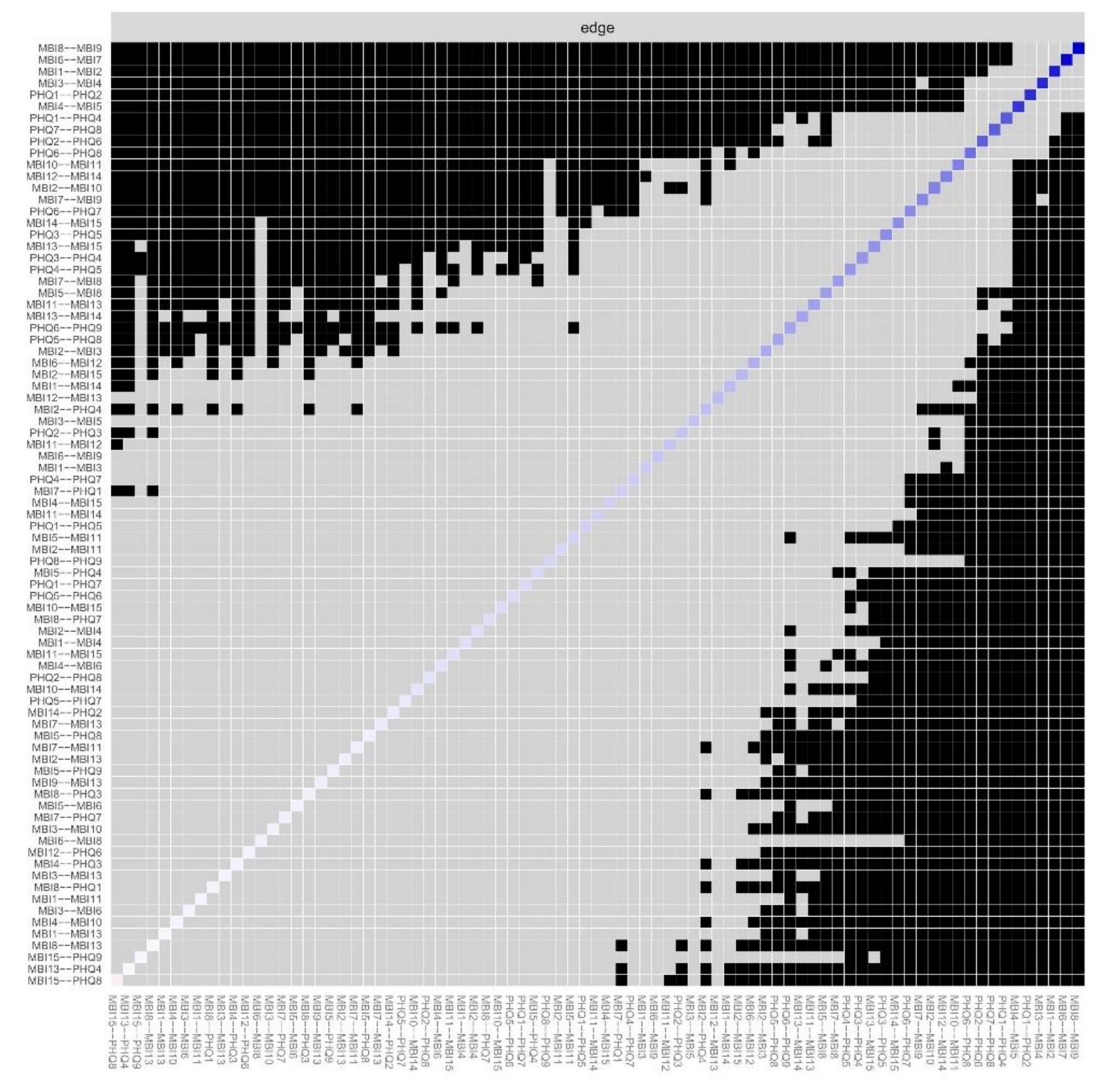
Estimation of edge weight difference by bootstrapped difference test Bootstrapped difference test for edge weights. The black box indicates that edge weights of the two corresponding variables have a significant difference (*P* < 0.05). The gray box indicates no significant difference (*P* > 0.05)

**Fig. 6:**
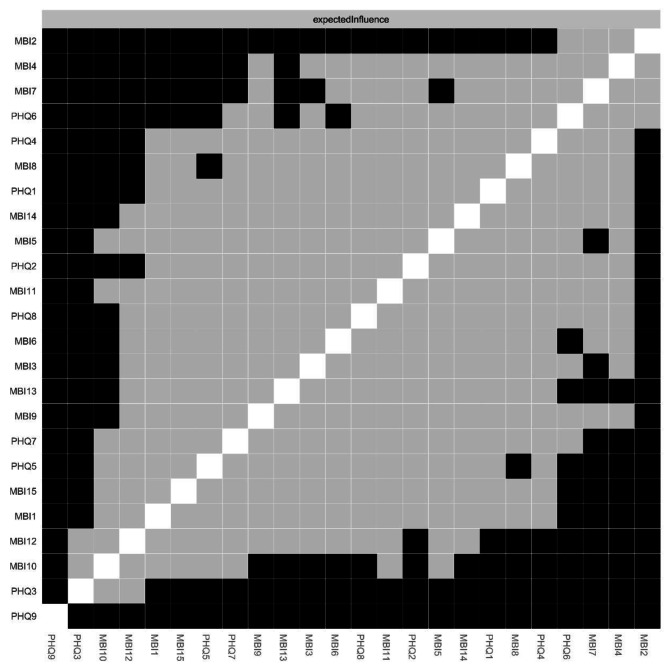
Nonparametric bootstrapped difference test Bootstrapped difference test for node expected influences. The black boxes indicate node expected influences that do differ significantly from one another (*P* < 0.05), while the gray boxes indicate node expected influences that don?t differ significantly (*P* > 0.05)

The network between burnout and depression showed a high excellent level of stability (Fig. 4). Both CS coefficients of EI and BEI were 0.68, which suggested that when 68% of the sample was dropped, the structure of the network did not change significantly. Supplementary Fig. S1 showed the bootstrapped 95% CI of edges and bootstrapped differences of edge weights, which were narrow and suggested high accuracy. Figure 5 showed the difference test of edge weights. The bootstrapped difference test found the most comparisons between EI were significantly different from the others (Fig. 6).

## Discussion

To our best knowledge, this was the first study to construct network model of burnout and depressive symptoms among ICU nurses at the late stage of COVID-19. The mean age of the participants was 28 years. The finding was similar to a cross-sectional survey showing the age of 26 years in ICU nurse of China [54], but lower than the average age of 39 years and 44 years reported in Italy and USA, respectively [55, 56]. Furthermore, WHO reported nurses with an average aged of 41 to 50 are the main force in this team from an international study of 106 countries [57]. The discrepancy for the difference may be due to lacking promoting professional development and leadership opportunities of nurses in China at present, resulting in shifting to administrative units in hospitals for senior nurses, such as those involved in nutrition, laundry positions, etc. [58].

The score of burnout symptoms of ICU nurses indicated that the prevalence of burnout was 48.2%, which was in keeping with the prevalence (45%) of burnout reported by a meta-analysis in ICU nurses during COVID-19 [15]. Besides, participants reported a high prevalence (64.1%) of depressive symptoms. It was similar to the depression rate (65.5%) of ICU nurses reported in a study with a structural equation model during COVID-19 pandemic [59]. The findings implied that it is essential to pay attention to the mental problems of this special population. Furthermore, it is suggested to allocate human resources based on their psychological conditions for hospital management personnel.

In the burnout symptoms, the three highest relations were “Indifferent” - “Doubt significance”, “Less interested”- “Less enthusiastic” and “Exhausted”- “Used up”. The finding was consistent with our previous research used network analysis discussing the associations between burnout and neuroticism. For “Indifferent”-“Doubt significance” and “Exhausted”-“Used up” in our network models, Chen et al. reported the strong relations of “Exhausted”-“Used up” and “Contributing”–“Good at the job” in exploring the connections between burnout and mental health among medical staff [60].The former was consistent with our studies. The results implied the heavy psychological burden among ICU nurses during COVID-19. The latter difference could be explained for issues such as lower social status of ICU nurses and insufficient respect from patients and society relative to medical counterparts [61, 62], and thus they were indifferent for contribution and doubted the significance of nursing occupation. For “Less interested”-“Less enthusiastic”, lack of interest toward work was associated with decreased enthusiasm during caring for patients [63].

In the depressive symptoms, the three highest relations were “Sad mood”-“Anhedonia”, “Fatigue”-“Anhedonia” and “Motor”-“Concentration”, which were in according with the findings of previous studies exploring the interrelationships between depression and other variables in medical staff during the COVID-19 pandemic [64–66]. However, one study found the relation between “Concentration” and “Suicide” weighed the highest [67]. The inconsistent result could be explained for different stages and professions. For the late stage of the epidemic, strict public health measures were canceled and healthcare workers saw the hope to conquer COVID-19, which gave rise to the stronger relationships between “Anhedonia” and “Sad mood” comparing to “Concentration” and “Suicide” in the network. For “Sad mood”-“Anhedonia” and “Fatigue”-“Anhedonia”, although our study conducted at the late stage of COVID-19, the mental and physical burden achieved highest because of sudden increased patients in ICU, which led to high levels of fatigue and then gave rise to feeling of anhedonia [68]. Besides, many ICU nurses also suffered from cough and fever owing to effecting by COVID-19 and had to keep working in their station because large number of patients were needed to care, which made nurses ICU having a sad mood, and caused anhedonia. As to “Motor”- “Concentration”, overload work and lack of communication with ICU patients led to showing psychomotor symptoms and lacking concentrations when caring patients in ICU nurses.

Within the depression-burnout symptoms, “Fatigue”-“Used up”, “Anhedonia”-“Less enthusiastic” and “Fatigue”-“Breakdown” weighted the strongest associations. For “Fatigue”-“Used up” and “Fatigue”-“Breakdown”, it was obvious fatigue was significantly related with emotional exhaustion (i.e., “Used up” and “Breakdown”). The relevant review in nurses reported the correlation of emotional exhaustion dimension in burnout was highest compared to others [27]. Furthermore, one literature suggested emotional exhaustion prevention should be paid more attention to relieve the fatigue of individuals, which could be achieved by better worktime and shift planning [69]. For “Anhedonia”-“Less enthusiastic”, excessive workload, such as irregular working hours, voluntary overtime, and closed contact with patients in ICU made nurses lose interest and enthusiastic in work tasks, thus to increase their inactive in working [8].

Expected influence (EI) of nodes performed well in recognizing specific symptoms that contributed strongly to the whole psychopathology symptom network. In this study, “Used up”, “Stressed” and “Less enthusiastic”, displayed the high EI in burnout-depression network. It meant these symptoms were critical and influential to understand the structure in burnout and depression model. A study reported a high risk of emotion exhaustion (38%) among ICU nurses in Belgium during pandemic [32], and this rate of emotional exhaustion in ICU nurses was more serious than other departments [70]. Furthermore, studies exploring the relations between burnout and depression showed that the correlation between emotional exhaustion and depression was higher compared to other relations [27]. The primary factors came from a higher ratio of patient-to-nurse in ICU than standard contract, prolonged working hours, and risks of transferring the infection to family members [71], which increased the risk of emotional exhaustion in ICU nurses. Regarding the above identified symptoms, some approaches were suggested. For example, establishing a reward system within ICU to ensure all nurses are rewarded and paid for their work equally [72]. Other strategies, such as enriching oneself, work-life balance schedule, and relaxed activity will be beneficial in reducing emotional exhaustion among ICU nurses [73, 74]. Furthermore, among those symptoms, the symptoms of “Used up” and “Stressed” were emotion exhaustion dimension of burnout. We have found “fatigue” was significantly related with emotional exhaustion in the burnout-depression symptoms. Thus, taking intervention targeting the symptom of “fatigue” will be effective to reduce the severity of burnout and depression symptoms of ICU nurses.

Predictability in the network model is used to indicate to what extent the variation of a node can be predicted by the variation of its connected nodes. The average predictability identified in each node reached 0.67, which suggested on average of 67% of variance of each node could be explained by their neighbor nodes. Thus, symptoms of “Used up”, “Stressed” and “Less enthusiastic” discovered in this study spotlighted the psychiatric health of ICU nurses.

For bridge symptom in the current network, the highest bridge expected influence was “Fatigue”, followed by “Breakdown” and “Used up”, indicating that these symptoms were critical to maintain the entire network model and target for intervention [37]. Previous literature had reported that symptom of “Fatigue” was a bridge symptom in relevant network analysis [24]. Suffering from fatigue was common among medical staff during the COVID-19 pandemics [75], and might resulted from high workload pressure and fear of contagion [75, 76]. “Breakdown” and “Used up” were identified as other key bridge symptoms. Maybe because these two symptoms were the consequence of “fatigue”. The evidence came from that edges of “Fatigue”-“Used up” and “Fatigue”-“Breakdown” showed highest correlations in the burnout-depression symptoms. It was well known that stress disorders had always been more prevalent among ICU nurses [77, 78]. Nurses working in ICU needed to copy with complicated and critical situations quickly and accurately [79, 80], and they also encountered much moments with separating and death than other department nurses in hospitals, which could further worsen their mental and physical fatigue. Especially, as the Chinese government lifted the restrictions implemented for “Zero-COVID” policy at the late stage of COVID-19, the number of severe COVID-19 patients were sent to ICUs for treatment, which placed extremely huge burden and overwhelmed nurses in ICU. Therefore, the current mental disorders in ICU nurses were worse than ever before.

Previous studies have shown that nurses working in specialized units such as ICU suffered from high levels of psychological and psychical tiredness [81]. Hence, interventions targeting “fatigue” of ICU nurses might reduce the severity of related symptoms. Related psychological interventions can improve the fatigue effectively, such as cognitive behavior therapy (CBT) [82], which is viewed as the first line of intervention thanks to its availabilities and effectiveness [83]. Besides, the nurse leaders can alleviate “fatigue” by shortening the shift length and overtime work of nursing staff during COVID-19 [84], and thus to lower the high level of burnout and depression among ICU nurses. It will be economic to design and implement related courses of fatigue and mental health during the initial and continuing education for nurses for ministry of education in China, which can help nurses identify and take timely intervene for fatigue symptom.

In general, exploring the highest centrality and bridge symptoms in the burnout and depression network was beneficial to take targeting interventions, and have far-reaching implications for reducing, identifying and prevention burnout and depression in ICUs nurses. Although the COVID-19 maybe has weakened in many countries, the infectious disease will never disappear in the world. Therefore, the current study provided advices or new thought to prevent and relieve the mental problems for nurse in ICU.

So far as we knew, this was the first study to visualize the relations between burnout and depression symptoms via network analysis among ICUs nurses in China at the late stage of COVID-19. However, some limitations should be noted. First, the causal relationships couldn’t be assessed as a result of a cross-sectional study. Second, the central symptoms and bridge symptoms identified in this study may not be generalized to other healthcare workers. Third, for the risk of contagion during the pandemic and closed management in ICUs, the data were collected by self-report measures by electronic questionnaires, which may cause bias.

## Conclusion

Despite the constraints above, the present study used network analysis to explore the complex relationship between burnout and depression in ICU nurses. The prevalence of burnout and depressive symptoms were high. The symptom of PHQ4(Fatigue) of depression was the bridge to connect the emotion exhaustion of burnout. The finding helps us to detect mental problems more effective and provides potential target for intervention for mental disorders in ICU nurses. Further studies are expected to monitor the fatigue quantitatively and explore personalized interventions based on the level of fatigue and in ICU nurses.

### Electronic supplementary material

Below is the link to the electronic supplementary material.

Supplementary Material 1

## Data Availability

The data that supported this research was available and can be obtained by from the corresponding authors. For the protection of privacy and ethics restriction, the data cannot be public available.

## Abbreviations

NA: Network analysis
MBI-GS: Maslach Burnout Inventory-General Survey
PHQ-9: 9-item Patient Health Questionnaire
WHO: World Health Organization
ARDS: Acute respiratory distress syndrome
ICU: Intensive Care Units
BOS: Burnout syndrome
LASSO: Least absolute shrinkage and selection operator
EBIC: Extended Bayesian information criterion
EI: Expected influence
BEI: Bridge expected influence
CS-C: Correlation stability coefficients
CBT: Cognitive behavior therapy
