# Supplementary material for: Relationship between depression and burnout among nurses in Intensive Care units at the late stage of COVID-19: a network analysis
Source: BMC Nurs. 2024 Apr 1;23:224. doi: 10.1186/s12912-024-01867-3 (PMC10983623; doi:10.1186/s12912-024-01867-3)

Figure S1. Bootstrapped confidence intervals of edge weights

Figure S1. Bootstrapped confidence intervals of edge weight

Accuracy of edge weights. The red line depicts the sample edge weights and the gray bar depicts the bootstrapped confidence interval.


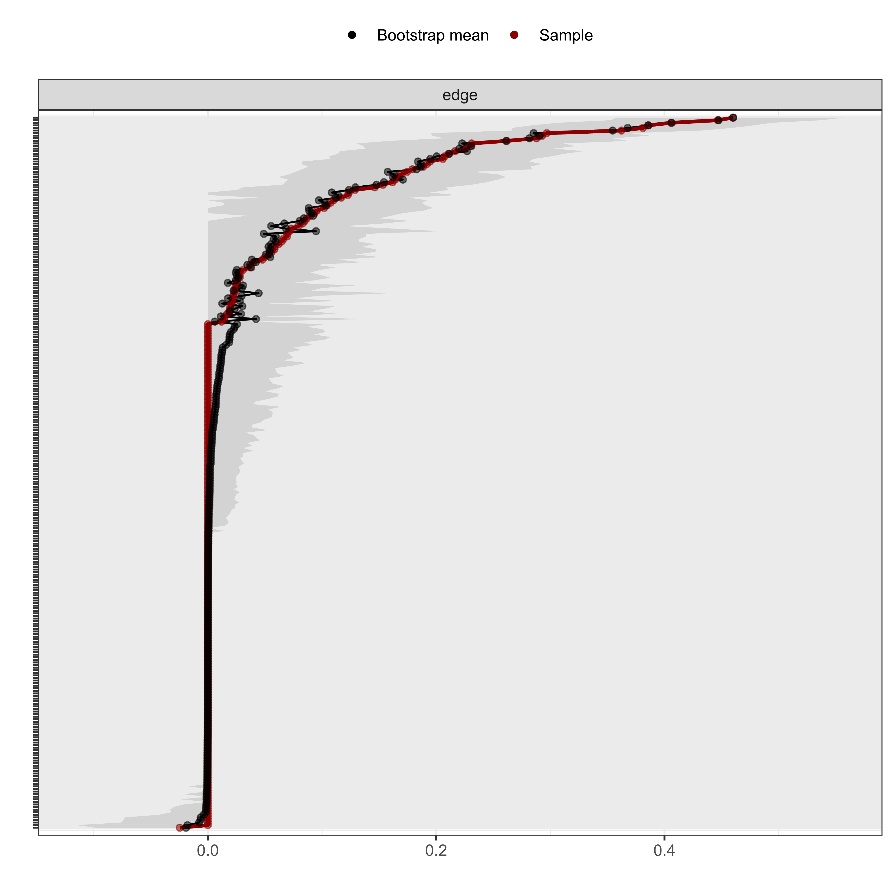

Supplement: Supplementary file 1 — Supplementary Material 1 [file 12912_2024_1867_MOESM1_ESM.docx]
